# Dysbiosis in Inflammatory Bowel Disease and Spondyloarthritis: Still a Long Way to Go?

**DOI:** 10.3390/jcm13082237

**Published:** 2024-04-12

**Authors:** Maria Consiglia Bragazzi, Federica Pianigiani, Rosanna Venere, Lorenzo Ridola

**Affiliations:** Department of Medical-Surgical Sciences and Biotechnology, Sapienza University of Rome, Polo Pontino, 04100 Rome, Italy; mariaconsiglia.braga@uniroma1.it (M.C.B.); federica.pianigiani@uniroma1.it (F.P.); rosanna.venere@gmail.com (R.V.)

**Keywords:** inflammatory bowel disease, spondyloarthritis, dysbiosis, microbiota, fecal microbiota transplantation, tumor necrosis factor alpha inhibitor

## Abstract

The association between Inflammatory Bowel Disease (IBD) and Spondyloarthritis (SpA) has been known for years, as has the concept that IBD is associated with an altered intestinal bacterial composition, a condition known as “dysbiosis”. Recently, a state of intestinal dysbiosis has also been found in SpA. Dysbiosis in the field of IBD has been well characterized so far, as well as in SpA. The aim of this review is to summarize what is known to date and to emphasize the similarities between the microbiota conditions in these two diseases: particularly, an altered distribution in the gut of *Enterobacteriaceae*, *Streptococcus*, *Haemophilus*, *Clostridium*, *Akkermansia*, *Ruminococcus*, *Faecalibacterium Prausnitzii*, *Bacteroides Vulgatus*, *Dialister Invisus*, and *Bifidubacterium Adolescentis* is common to both IBD and SpA. At the same time, little is known about intestinal dysbiosis in IBD-related SpA. Only a single recent study has found an increase in Escherichia and Shigella abundances and a decrease in Firmicutes, Ruminococcaceae, and Faecalibacterium abundances in an IBD-related SpA group. Based on what has been discovered so far about the altered distribution of bacteria that unite both pathologies, it is appropriate to carry out further studies aiming to improve the understanding of IBD-related SpA for the purpose of developing new therapeutic strategies.

## 1. Introduction

The term “gut microbiota” refers to commensal and pathogen bacteria, and other microorganisms that colonize the intestinal tract. The gut of healthy people is dominated by *Firmicutes* and *Bacteroidetes*, but also, to a lesser extent, *Proteobacteria*, *Actinobacteria*, and *Verrucomicrobia* [1]. Despite inter-individual variability, the functionality of the microbiota in healthy patients is preserved as long as the state of “eubiosis” (by which we mean the balance between bacterial species) is maintained [2,3]. When the balance fails, qualitatively and/or quantitatively, we speak of dysbiosis [4]. In dysbiosis, the functions performed by bacteria are impaired, resulting in a loss of integrity of the gut barrier. This facilitates the translocation of bacteria and an increased activation of the inflammatory state [5].

In this review, we analyze which alterations of the intestinal microbiota in patients with Inflammatory Bowel Disease (IBD) and those with Spondyloarthritis (SpA) are known to date, trying to highlight the similarities between the microbiota conditions in these two conditions and their possible implications.

SpA is a group of inflammatory joint diseases. These diseases have similar clinical and genetic features, such as association with the HLA-B27 antigen and involvement of the axial skeleton and peripheral joints.

Unlike rheumatoid arthritis (RA), SpA is mostly seronegative because it does not show the presence of autoantibodies [6]. In accordance with the Assessment of Spondyloarthritis International Society (ASAS), based on the clinical presentation, SpA is divided into predominantly axial SpA (axSpA) where the inflammation primarily affects the joints of the spine, thoracic cage, and pelvis, and peripheral SpA where the inflammation affects the peripheral joints (asymmetrical oligoarthritis, enthesitis, and dactylitis) mainly of the lower limbs.

axSpA includes two subgroups: non-radiographic axial SpA (nr-axSpA) without radiographic sacroiliitis and radiographic axial SpA (r-axSpA)** [7]**. SpA also often presents common extra-musculoskeletal manifestations and may be associated with other autoimmune-related diseases including chronic Inflammatory Bowel Disease (IBD-related SpA). IBD is a chronic immune-mediated disease of the digestive tract that is highly heterogeneous and debilitating, sometimes characterized by relapses and worsening. They include Crohn’s disease (CD), ulcerative colitis (UC), and unclassified IBD (IBD-U). IBD-U is a form of IBD of the colon whose characteristics make it impossible to define CD or UC colitis at the time of diagnosis [8,9,10,11]. Regarding IBD-related SpA, it can be axial or peripheral [12]. Generally, the latter are more common in CD and in the female gender, while axial spondylitis occurs indistinctly in CD or UC and in both sexes [12,13]. Over the years, the hypothesis that there is a close correlation between the gut and joints has always been more corroborated, and, for this reason, the so-called “gut–joint axis” has become an emerging research area in SpA [14,15,16]. At the same time, it has been known for years that IBD is associated with an altered intestinal bacterial composition, a condition referred to as intestinal dysbiosis. Recently, several studies have also described a state of intestinal dysbiosis in a kind of r-axSpA: ankylosing spondylitis (AS) [17,18,19,20]. While gut dysbiosis has been recognized in patients with AS, the microbiota showed no statistically significant differences between patients with non-radiographic axSpA (nr-axSpA) and healthy controls. Despite this, in the overall axSpA group (nr-axSpA and AS combined), the presence of gut dysbiosis was associated with worse disease activity [21,22,23]. To date, therefore, little is known about the gut microbiota in patients with non-radiographic axSpA (nr-axSpA) [24].

## 2. IBD-Related SpA

The association between IBD and SpA is frequently reported [12,13]. It has been observed that 5–7% of patients with SpA developed IBD, while 13% of patients with IBD develop SpA [25]. About 60% of axSpA patients have evidence of subclinical gut inflammation (which is identified by histological evidence of microscopic gut inflammation in the absence of overt gastrointestinal symptoms), and 5–10% develop diagnosed IBD [26]. Furthermore, it has been shown that the appearance of subclinical intestinal inflammation occurs in all known subsets of SpA. It has been observed that, in cases of remission of joint inflammation, there is even a disappearance of intestinal inflammation [27,28].

## 3. Gut–Joint Axis

Gastrointestinal inflammation has been associated with SpA for many years. In particular, in a publication as early as 1958, it was described how, out of 222 cases of patients affected by AS, 5 of these patients had concomitant IBD. Simultaneously, the authors highlighted that in the same years, other articles concerning the correlation between these two pathologies showed a similar incidence. From this, the first hypothesis of correlation between these two diseases was formulated [29]. In subsequent years, some elements emerged that corroborated these initial observations. Since then, based on this hypothesis, several studies have been conducted and several genetic and immunological factors have been identified that play an important role in both gut and joint inflammation. These include, for example, the increased expression of IL-23, which promotes the activation of group 3 innate lymphoid cells (ILCs, a subset of natural killer cells), resulting in the expression of the cytokines IL-17 and IL-22 [30,31]. Among genetic factors, polymorphisms of genes in the IL-17 and IL-23 axis (IL23R, IL12B, STAT3, JAK2, PTGER4, PUS10, and IL18RAP) shared by both diseases, SpA and IBD, could power this immune pathway [32].

**Figure 1:**
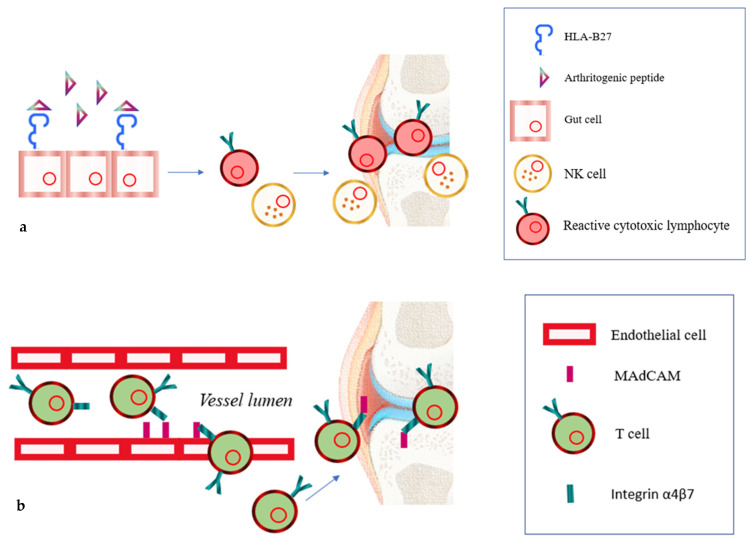

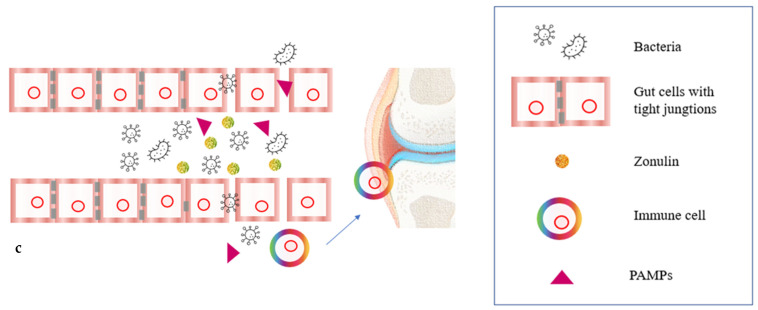
Three probable mechanisms that could explain the link between joint inflammation and the gut. First hypothesis: the surface molecule HLA-B27, interacting with the “arthritogenic peptide” in the gut, would lead to the activation of cytotoxic lymphocytes and NK cells whose activity would then be expressed in the joints (**a**). Second hypothesis: Activated T-cells in the gut overexpress adhesion molecules such as α4β7 integrin on their surface, which, through binding to MAdCAM, promotes T-cell homing in gut-associated lymphoid tissues. However, even in joints, the endothelium expresses MAdCAM, favoring T-cell homing there as well (**b**). Third hypothesis: exposure to large amounts of bacteria in the gut leads to the secretion of zonulin, which causes disassembly of the ZO-1 protein from the tight junction complex, resulting in increased intestinal permeability with an inability to prevent the translocation of bacteria and their products; this follows an activation of the immune system, which, therefore, would transmigrate from the intestine to the joints (**c**).

Thus, the concept of the “gut–joint axis” was born. To explain this relationship, three hypotheses have been proposed. These mechanisms are not mutually exclusive but can also coexist. The first hypothesis revolves around the role of HLA-B27, a surface molecule belonging to MHC type 1, whose presence is strongly associated with SpA (96% of patients with SpA are carriers of HLA-B27, while only 7–12% of healthy controls are carriers) [33]. This would seem to be responsible for the presentation of the so-called “arthritogenic peptide”, with the consequent activation of cytotoxic lymphocytes and NK cells. These activated cells could play a role in the pathogenesis of the joints [34]. The nature of the “arthritogenic peptide” is unclear, but its microbial or infectious origin is most likely and is supported by the observation that enteric bacterial infections sometimes precede SpA. Based on this, the match with HLA-B27 and the “arthritogenic peptide” could occur in the gut (Figure 1a) [35,36].

The second hypothesis concerns abnormal T-cell trafficking. The activation of these immune cells in the gut causes an increased expression of adhesion molecules on the surface of gut cells, such as α4β7. α4β7 is an integrin responsible for T-cell homing in gut-associated lymphoid tissues. T-cell homing occurs thanks to the binding of α4β7 to the mucosal cell adhesion molecule addressin (MAdCAM), which is present on the high endothelial venules of mucosal lymphoid organs [37]. MAdCAM is also found expressed on various joint endothelial cells; as a result, immune cells from the gut would enter the joints, triggering an inflammatory process there too [38,39]. Corroborating this, in patients with SpA, identical T-cell clones were found in their joints and gut (Figure 1b) [40].

The last factor characterizing the gut–joint axis is the structural change of the intestinal barrier because of gut dysbiosis [41,42]. In healthy conditions, intestinal epithelial cells, in addition to promoting the absorption of water and nutrients, also play an important role in generating physical and chemical barriers to protect the mucosa from commensal and pathogenetic microorganisms. This barrier is made by a mucosal layer that acts as both a chemical and physical barrier [43]. In particular, the chemical barrier function is mainly carried out in the small intestine, while the physical barrier function is mainly exercised in the large intestine [43]. The chemical barrier is made up of AMPs, Reg3γ, lysozyme, and secretory phospholipase A2 that are produced by Paneth cells; the physical barrier consists of tight junctions between the enterocytes, glycocalyx on the microvilli of enterocytes, and a double layer of mucus. The latter, made up of Mucin2 protein that is produced by the goblet cells, is loose on the outside, where it is populated by Lactobacilli and Bifidobacteria, and compact on the inside, where it is devoid of bacterial colonization and where some antimicrobial molecules have been found (e.g., immunoglobulin A) [44,45,46,47]. Tight junctions between cells are not static but rather dynamic complexes [48]. The regulatory mechanisms that cause changes in these structures are still not entirely clear, except for zonulin, an intestinal permeability modulator [49]. The exposition of large amounts of bacteria leads to zonulin secretion, which causes the disassembly of the protein ZO-1 from the tight junctional complex, with a consequent increase in permeability and the transmigration of immune cells from the gut to joints (Figure 1c) [50]. The increase in permeability, normally, represents a defensive mechanism that leads to the activation of the innate immune response when there is bacterial overgrowth or dysbiosis [49,50]. The concomitant presence of environmental triggers and genetic predispositions could turn this physiological defense mechanism into a pathological one [49,50].

## 4. Gut Microbiome in Healthy People

In the past, understanding of the gut microbiome was limited because microbiological culture was the only way to study its composition. Later, culture-independent techniques were developed. These make it possible to identify nonculturable microorganisms quickly and accurately and to compare the variety and relative abundance of microbial taxa [51].

As a result, multiple studies have emerged, such as the “Metagenomics of the Human Intestinal Tract” (METAHIT). Based on the predominant taxa, researchers in the METAHIT project identified three so-called “enterotypes”: type 1, type 2, and type 3. Type 1 is characterized by a prevalence of Bacteroides, which derive energy mainly from carbohydrate and protein fermentation; type 2 is characterized by the prevalence of *Prevotella*, and type 3 by the dominance of *Ruminococcus* [52]. Methanogenic archaea (primarily Methanobrevibacter smithii), *Eucarya* (predominantly yeasts), and multiple phages are also present in the guts of healthy people [53]. Even though it can be classified into these enterotypes, the microbiota remains unique in everyone [2]. In dysbiosis, the functions performed by the bacteria are compromised and the integrity of the intestinal barrier is lost [54,55]. This causes increased permeability and an inability to prevent bacterial translocation and their products (also called pathogen-associated molecular patterns, or PAMPs), as well as the reabsorption of damage-associated molecular patterns (DAMPs) [5]. These molecules lead to increased activation of M1 and M2-type macrophages. In addition, these reduce the expression of regulatory T-cells (Treg), which under normal conditions suppress hyperactivation of the immune system. This leads to increased Th1 and Th17 levels with chronicization of the inflammatory state [5].

## 5. Microbiota Alterations in IBD

To date, it is known that genetic, immune, and environmental factors contribute to the development of IBD; among the environmental factors, a central role is played by the gut microbiota [56]. In fact, it has been observed that patients with active disease are characterized by some differences compared to healthy people in the bacterial diversity, composition, and/or abundance of their microbiota [57]. In particular, a decrease in *Firmicutes* and a concomitant increase in *Proteobacteria* and some members of *Bacteroidetes* were observed on tissue samples from patients with active IBD [57]. Gophna et al. identified these variations in CD patients but not in UC [58]. An increase in mucolytic bacteria, such as *Ruminococcus Gnavus* and *Torques*, was detected in both tissue and fecal samples in UC and CD patients; these increased the penetration of pathogens into intestinal tissues [59,60,61]. Other authors [62,63,64,65,66,67] have identified on intestinal tissue samples additional variations in the distribution of bacteria in patients with IBD: in particular, there is a concordance in the finding of an increased concentration of *Enterobacteriaceae* and *Fusobacteriaceae* and a reduced presence of *Ruminococcaceae* and *Faecalibacterium* (which would appear to be correlated with the progression of colorectal carcinoma); also less homogeneously described is an increase in the following families, *Veillonellaceae*, *Neisseriaceae*, *Pasteurellaceae*; a reduction in the following orders, *Erysipelotrichales*, *Bacteroidales*, and *Clostridiales*; and a reduction in the following taxa, *Roseburia*, *Phascolarctobacterium*, *Dorea*, *Blautia*, *Collinsella*, *Ruminococcus*, *Akkermansia*, *Coprococcus*, and numerous other taxa within the families of *Lachnospiraceae*; these changes correlate strongly with disease activity. Among these, *Faecalibacterium Prausnitzii*, a member of *Firmicutes*, has shown a powerful anti-inflammatory effect: in patients with CD undergoing surgical resection, a reduction in its presence in stool samples correlates with a high risk of disease recurrence within 6 months [68,69]. Gevers et al. [66], in addition to agreeing with the microbial alterations in intestinal tissue samples of IBD pediatric patients evidenced above, highlighted how the microbiota in stool samples from patients with IBD is also characterized by an increased presence of sulfate-reducing *Gammaproteobacteria* and *Deltaproteobacteria, Streptococcus, Lactobacillus, Enterococcus, Megasphaera, Campylobacter, Bacteroides fragilis*, *Proteus mirabilis*, and *Klebsiella pneumoniae*, as well as a lower presence of *Dorea, Neisseriaceae, and Fusobacteriacea.* Other authors [70,71,72,73], again on stool samples, have also observed a decrease in *Bifidobacterium longum* and *Eubacterium*, with an increase in *Actinomyces* spp., *Intestinibacter* spp., and *Escherichia coli*. Thus, it was observed that dysbiosis at the tissue level is lower than that observed in stool samples [63]. The result, anyway, is an overall loss of microbial diversity [74]. These alterations in the stability and diversity of the microbiota can lead to a reduction in functions that the bacteria perform in cooperation with the host; this can cause, if we consider the way the microbiota promotes the increase in the intestinal barrier that we analyzed earlier, a reduction in the gut barriers, favoring and amplifying inflammatory processes locally and systemically [75]. The studies focus, predominantly, on bacterial alterations of the intestinal flora, but there is some evidence that alterations in fungi, bacteriophage, and *Archaea* may also be involved in the etiopathogenesis of IBD: the virome and the mycobiota also exhibit a reduction in diversity in these patients [76]. Regarding the gut virome, patients with IBD are characterized by an expansion of *Caudovirales bacteriophages* in fecal samples, and an increase in *Pneumoviridae* and *Herpesviridae* families, with a decrease in the *Anelloviridae* family in tissue samples [77,78,79]. Additionally, mouse models demonstrated that *Norovirus* infection contributes to the development of intestinal inflammation [80]. Regarding the mycobiota, instead, a decreased abundance of *Saccharomyces Cerevisae* and *Candida Tropicalis* and a simultaneous increase in *Candida Albicans*; *Aspergillus; Wallemia; Epicoccum* spp. in stool samples; and *Malassezia Restricta*, a commensal skin yeast, in tissue samples and mouse models have been observed, but it is not clear what the real contribution is of these alterations in IBD [81,82,83,84] (Table 1).

## 6. Microbiota Alterations in SpA

Several studies have described an alteration in the gut microbiota also in SpA, although there is much discordance between these studies [17,18,87,89]. In a study, Costello et al. [18] took terminal ileum biopsies from 9 AS cases naïve to TNF inhibitors and with an onset less than 48 months and compared them with biopsies taken from 9 healthy control patients. Terminal ileal biopsies from patients with AS had a higher abundance of *Bacteroidaceae*, *Lachnospiraceae*, *Porphyromonadaceae*, *Rikenellaceae*, and *Ruminococcaceae*, with lowered *Prevotellaceae* and *Veillonellaceae* compared with healthy controls. At the same time, in a study by Tito et al. [24], an increase in *Dialister Invisus* was observed on intestinal mucosa samples from 27 patients with newly diagnosed SpA compared with 15 healthy controls, and this correlates with disease status. The latter information disagrees with what was later observed on an analysis conducted on fecal samples, by Yin et al. [20] who, comparing shotgun metagenomic sequencing on stool from 127 cases of SpA and 123 healthy controls, instead described a reduction in *D. Invisus*. In the same study, it was also observed that, in the SpA, there was an increase in the bacteria *Clostridiales 1 7 47FAA, Clostridium Boltae*, and *Clostridium Hathewayi*, while *Bifidobacterium Adolescentis*, *Coprococcus Comes, Lachnospiraceae 5 1 63FAA, Roseburia Inulinivorans*, and *P. copri* were depleted. Then, Wen et al. [17] collected fecal samples from 97 patients with AS and 114 healthy controls, and they found that *Bifidobacterium, Prevotella melaninogenica*, *Prevotella copri*, and *Prevotella* sp. C561 could participate in the development process of AS, stimulating an immune reaction that then targets joint tissues. Next, Zhou et al. [87] collected and compared the fecal metagenome of 85 untreated AS patients with 62 healthy controls by shotgun metagenomic sequencing and 23 post-treatment feces of these AS patients. They also observed an enrichment of some bacterial species in AS patients, although different from those previously described by Wen et al. [17]. The fecal metagenome of patients with AS was richer in *Bacteroides coprophilus*, *Parabacteroides Distasonis*, *Eubacterium Siraeum*, *Acidaminococcus Fermentans*, and *Prevotella copri*, and it had a reduction in *Ruminococcus Obeum* and several species capable of producing SCFA, such as *Eubacterium Hallii*, *Coprococcus Catus*, *F. Prausnitzii*, and *Coprococcus Eutactus*. Previously, Scher et al. [89], through the use of 16S ribosomal RNA sequencing on stool samples, also observed a lower abundance of *Ruminoccoccus* and *Akkermansia* in the gut microbiota of 16 patients with psoriatic arthritis (PsA) than that observed on stool samples from 15 patients with skin psoriasis and 17 healthy controls. Later, Zhang et al. [22], also analyzing 207 fecal samples from 103 subjects with SA and 104 healthy controls, found that *Ruminococcus* was significantly lower in SA than in samples from healthy controls. In the same study, they also noted a reduction in *Lachnospira*, *Clostridium IV*, and *Clostridium XlVb* and an enrichment of *Actinobacteria*, *Megamonas*, *Sutterella*, *Dorea*, *Blautia*, and *Bacteroides Plebeius*. Relative to *Prevotellaceae*, in contrast to the data observed by Costello et al. [18] on ileal biopsies, Zhang et al. [22] found no significant diversity on fecal samples from patients with SA compared with those from healthy controls. More recently, Marquez-Ortiz et al. [86] analyzed the gut microbiota from 32 patients with SpA and 7 healthy controls, by colonoscopy aspiration lavages (CALs) and fecal samples. In patients with SpA, an increase in the *Enterobacteriaceae* family, *Succinivibrio* spp., and *Prevotella stercorea* and a significant decrease in SCFA producers *Coprococcus catus* and *Eubacterium biforme* were observed (Table 1).

## 7. Microbiota Alterations in IBD and SpA Compared

Currently, there are a few studies that compare microbiota alterations in IBD and SpA [21,24,88]. Even less explored is the field of IBD-related SpA [19]. Sternes et al. [21], using a combination of stool samples and biopsies of the terminal ileal mucosa, colon, and rectum, compared the microbiota of 33 patients with AS (including 5 AS-IBD), 59 IBD patients, and 105 healthy controls. The gut microbiota of AS and IBD patients is significantly different from the microbiota of healthy controls, but many of the differentially abundant genera were not consistent between AS and IBD except for the enrichment of two potentially pathogenic genera, *Haeomophilus* and *Streptococcus*; it follows that a few pathogenic genera may be the common factor initially triggering these diseases. A direct role of the gut microbiota in triggering both SpA and IBD is highly supported by animal models. In HLA-B27 transgenic rats, the spontaneous development of arthritis and gut inflammation was prevented by rearing the animals under germ-free conditions; the colonization of germ-free rats with a limited number of bacteria (even with single bacterial species) triggered both gut inflammation and arthritis; specifically, *Bacteroides Vulgatus* would appear to have this capability [88]. Among the first to pay attention to the possible link between the microbiota, IBD, and SpA were Klingberg et al. [19]. Collecting stool samples, they compared the microbiota and fecal calprotectin levels in 105 patients with AS, 18 patients with UC, and 17 healthy controls [19]. They observed that patients with AS and elevated fecal calprotectin (≥200 mg/kg) show different microbiota alterations than those with AS and normal fecal calprotectin (≤50 g/kg), and particularly a reduction in *F. Prausnitzii* and *Clostridium* genera with an increase in the *Streptococcus* genus. The same changes were observed in patients with UC, suggesting a local interplay between intestinal microbiota and gut inflammation in AS [19]. They also showed an increase in *Enterobacteriaceae* in both UC and AS patients [19]. More recently, however, Zhangni et al. [85] included in their study seventy-three patients with UC: patients were divided into axSpA and non-axSpA groups. There were 14 patients with axSpA, with an incidence of 19.2%. In fecal samples, they have found that *Firmicutes, Ruminococcaceae,* and *Faecalibacterium* abundances were decreased, and *Proteobacteria* such as *Escherichia* and *Shigella* abundances were increased in the axSpA group compared with those of the non-axSpA group. They concluded that changes in the relative abundances of these bacteria may be related to the incidence of axSpA.

Differences have also been observed in studies conducted by taking intestinal biopsies. Tito et al. [24] observed that *Dialister* abundance is greater on histologic pieces of biopsies taken on inflamed colonic and ileal mucosa than on those taken on noninflamed colic or ileal mucosa from patients with AS (Table 1).

## 8. Effects of TNFi

The introduction of biological drugs, particularly TNFi, has revolutionized the treatment of IBD-related SpA. The 2019 recommendations of the American College of Rheumatology (ACR), the Spondylitis Association of America (SAA), and the Spondyloarthritis Research and Treatment Network (SPARTAN) encourage the use of TNFi monoclonal antibodies in IBD-related SpA patients. In particular, the use of Infliximab or Adalimumab presents lower risks of IBD exacerbations than other TNFi [90]. In this regard, however, it is important to specify that not all TNFi used in rheumatology are suitable for the treatment of IBD: for example, Etanercept is not approved for treating IBD patients; in contrast, Golimumab is given only for UC but not for CD. On the other hand, Certolizumab pegol is only approved for CD by the United States Food and Drug Administration (FDA) but not by the European Medicines Agency (EMA) [91].

It is known that treatment with TNFi could restore the gut barrier in IBD patients, while little is known about its effect on the gut microbiota [92]. Yin et al. [20], analyzing and comparing the stools of 123 healthy controls, 67 AS cases treated with TNFi, and 60 AS cases who have not received TNFi treatment, demonstrated that TNFi exerts a role in re-establishing eubiosis in patients with SpA, promoting a rebalancing of some species, including *P. Copri*, *F. Prausnitzii,* unclassified *Bilophila*, *K. pneumoniae*, *Ruminococcus Bromii*, and *Eubacterium Biforme.* Dai et al. [93] collected fecal samples from 11 healthy controls and 24 AS patients before and after treatment with TNFi and observed that the composition of some specific bacteria altered in AS patients can be restored to healthy controls after TNFi treatment, particularly SCFA-producing bacteria such as *Megamonas* and *Lachnoclostridium*. They also observed that the abundance of these bacteria was negatively correlated with disease severity, supporting the possible impact of microbiota on AS through their metabolites (e.g., SCFAs). In a recent study, under the hypothesis that TNFi can restore eubiosis in patients with IBD-related SpA, Ditto et al. [94] analyzed fecal samples from 20 drug-naïve patients with this disease treated with TNFi at baseline and after six months of therapy. They found the relative abundance of some species restored, with significant increases in *Lachnospiraceae*, *Clostridia*, *Lactobacillus*, *Coprococcus*, and *R. Gnavus* and decreases in *Proteobacteria* and *Gammaproteobacteria*. However, they showed no differences between responders and non-responders to treatment, suggesting that TNFi can restore the fecal microbiota regardless of clinical response [94]. Bazin et al. [95] instead analyzed fecal samples from 19 patients with SpA (excluding patients also suffering from IBD) before starting treatment with TNFi and after 3 months of therapy; firstly, they compared the fecal microbiota before and after treatment, confirming that for all patients, as well as for responder or non-responder subgroups, diversity seemed to be restored, but no significant differences were found in the concentration of any bacteria. Secondly, they looked for certain bacteria before TNFi therapy that could be predictive of the treatment outcome: they found that *Burkholderiales*, belonging to the *Betaproteobacteria* phylum, appeared higher before treatment in patients who were responders after 3 months; therefore, they hypothesized that it might be a predictor of response [95]. Vallier et al. [96], in contrast, analyzing stool samples of 61 patients with AxSpA (they also excluded IBD patients) before and after treatment with TNFi, observed that *Sutterella* and other genera belonging to the class *Clostridia* were present only in non-responder patients, suggesting the research of these bacteria as a novel non-invasive index that could predict the response to TNFi (Table 2).

## 9. Fecal Microbiota Transplantation

Based on what is known so far about gut dysbiosis in IBD and AS, the use of fecal microbiota transplantation (FMT) is, to date, an active field of research, with numerous studies emphasizing its short-term efficacy (data confirming its long-term efficacy are lacking) [97,98]. Although there are still many controversial aspects regarding safety and possible adverse effects that need further investigation, FMT remains a hope for IBD patients [97]. Instead, the use of this treatment in patients with SpA is not explored [99]. Recently, Wang et al. [99] reported the first promising case of treatment of a 24-year-old male patient with IBD-related AS that was refractory to conventional treatments, including tumor necrosis factor alpha inhibitors (TNFi); he underwent three FMTs. After FMT, they observed in the stool of the recipient an increase in the genera *Faecalibacterium* and *Parasutterella* and a decrease in *Escherichia*, *Shigella*, and *Intestinibacter*, which was correlated with a decrease in disease activity.

## 10. Conclusions

Over the years, the involvement of microbiota alterations in the onset of IBD and SpA, respectively, has been studied extensively; this has made it possible to identify certain bacteria whose relative abundance may contribute to both of these diseases separately. Up until now, it has emerged that the increased concentration in the gut of *Enterobacteriaceae*, *Streptococcus*, *Haemophilus*, *B. Vulgatus*, and *D. Invisus*, as well as the reduced presence of the taxa *Clostridium, Ruminococcus*, and *Akkermansia* and the species *F. Prausnitzii* and *B. Adolescentis* is common to both IBD and SpA. Instead, the field of IBD-related SpA does not seem to have been explored much. Only a single recent study has found an increase in *Proteobacteria* abundance (such as *Escherichia* and *Shigella*) and a decrease in *Firmicutes*, *Ruminococcaceae*, and *Faecalibacterium* abundances in an IBD-related SpA group. Interestingly, the altered levels of *Protebacteria, Ruminoccocaceae*, and *Faecalibacterium* abundance characterize IBD, SpA, and IBD-related SpA patients, while the altered abundance of *Firmicutes* is shared by IBD and IBD-related SpA patients. Therefore, as this is still a little-explored area, it would be interesting to conduct studies comparing the microbiota alterations of this population with those of individuals suffering exclusively from IBD or SpA. Furthermore, it remains to be understood what the therapeutic implications of identifying these alterations might be: targeted therapies and disease biomarkers are two future possibilities of the microbiota that need to be further investigated. An upcoming challenge is therefore to understand whether microbiota alterations may be more related to one disease condition than another; this could provide us with useful elements for early diagnosis. In addition, in these patients, the re-establishment of eubiosis could accelerate clinical remission. On this point, although the listed studies revealed changes in the microbiota induced by TNFi therapy, the concordance between the results obtained is currently minimal and further analyses would be necessary.

## Figures and Tables

**Table 1:** Changes in gut microbiota in IBD and SpA compared.

IBD		SpA			IBD-Related SpA
CD	UC		AS	PsA	
Increase					
***Proteobacteria*** **(F)(T)** [57,58,63]	***Proteobacteria*** **(F)(T)** [57,63]	*Actinobacteria* (F) [22]	*Actinobacteria* (F) [22]		*Proteobacteria* (F) [85]
*Bacteroidetes* (F)(T) [57,58,63]	*Bacteroidetes* (F)(T) [57,63]				
*Gammaproteobacteria* (F) [66] *					
*Deltaproteobacteria* (F) [66] *					
***Enterobacteriaceae*** **(T)(F)** [21,66] *	***Enterobacteriaceae*** **(T)(F)** [19,21]	***Enterobacteriaceae*** **(F)(CAL)** [21,86] ^#^	***Enterobacteriaceae*** **(F)(CAL)** [21]		
*Fusobacteriaceae* (T) [62]	*Fusobacteriaceae* (T) [62]	*Bacteroidaceae* (T) [18]	*Bacteroidaceae* (T) [18]		
*Veillonellaceae* (T) [66] *		*Lachnospiraceae* (T) [18]	*Lachnospiraceae* (T) [18]		
*Neisseriaceae* (T) [66] *		*Porphyromonadaceae* (T) [18]	*Porphyromonadaceae* (T) [18]		
*Pasteurellaceae* (T) [66] *		*Rikenellaceae* (T) [18]	*Rikenellaceae* (T) [18]		
		*Ruminococcaceae* (T) [18]	*Ruminococcaceae* (T) [18]		
***Streptococcus*** **(F)(T)** [21,66] *	***Streptococcus*** **(F)(T)** [21]	***Streptococcus*** **(F)(T)** [19,21]	***Streptococcus*** **(F)(T)** [19,21]		*Escherichia* (F) [85]
***Haeomophilus*** **(F)(T)** [66] *		***Haeomophilus*** **(F)(T)** [21]	***Haeomophilus*** **(F)(T)** [21]		*Shighella* (F) [85]
*Enterococcus* (F) [66] *		*Blautia* (F) [22]	*Blautia* (F) [22]		
*Campylobacter* (F) [66] *		*Dorea* (F) [22]	*Dorea* (F) [22]		
*Lactobacillus* (F) [66] *		*Bifidobacterium* (T) [17]	*Bifidobacterium* (T) [17]		
*Megasphaera* (F) [66] *		*Megamonas* (F) [22]	*Megamonas* (F) [22]		
		*Sutterella* (F) [22]	*Sutterella* (F) [22]		
*Ruminococcus Torques* (T) [59,61]	*Ruminococcus Torques* (T) [61]	*Prevotella melaninogenica* (T) [17]	*Prevotella melaninogenica* (T) [17]		
*Bacteroides fragilis* (F) [66] *		*Prevotella copri* (T)(F) [17,87]	*Prevotella copri* (T)(F) [17,87]		
*Proteus mirabilis* (F) [66] *		*Prevotella* sp. C561 (T) [17]	*Prevotella* sp. C561 (T) [17]		
*Klebsiella pneumoniae* (F) [66] *		*Prevotella stercorea* (CAL) [86]			
*Ruminococcus Gnavus* (T) [59,60,61]	*Ruminococcus Gnavus* (T) [60,61]	*Bacteroides coprophilus* (F) [87]	*Bacteroides coprophilus* (F) [87]		
***Bacteroides Vulgatus*** **(A)** [88]	***Bacteroides Vulgatus*** **(A)** [88]	***Bacteroides Vulgatus*** **(A)** [88]			
***Dialister Invisus*** **(T)** [24]	***Dialister Invisus*** **(T)** [24]	***Dialister Invisus*** **(T)** [24]			
*Actinomyces* spp. (F) [73]	*Actinomyces* spp. (F) [73]	*Bacteroides Plebeius* (F) [22]	*Bacteroides Plebeius* (F) [22]		
*Intestinibacter* spp. (F) [73]	*Intestinibacter* spp. (F) [73]	*Parabacteroides Distasonis* (F) [87]	*Parabacteroides Distasonis* (F) [87]		
*Escherichia coli* (F) [67]		*Eubacterium Siraeum* (F) [87]	*Eubacterium Siraeum* (F) [87]		
		*Acidaminococcus Fermentans* (F) [87]	*Acidaminococcus Fermentans* (F) [87]		
		*Clostridium Boltae* (F) [20] ^#^	*Clostridium Boltae* (F) [20] ^#^		
		*Clostridium Hathewayi* (F) [20] ^#^	*Clostridium Hathewayi* (F) [20] ^#^		
		*Clostridiales b. 1 7 47FAA* (F) [20] ^#^	*Clostridiales b. 1 7 47FAA* (F) [20] ^#^		
		*Succinivibrio* spp. (CAL) [86] ^#^			
	*Pneumoviridae* (T) [79]				
*Herpesviridae* (T) [78]	*Herpesviridae* (T) [78]				
*Caudovirales bacteriophages* (F) [77]	*Caudovirales bacteriophages* (F) [77]				
*Norovirus* (A) [80]	*Norovirus* (A) [80]				
*Epinococcus* spp. (F) [84]	*Epinococcus* spp. (F) [84]				
*Wallemia* (F) [84]	*Wallemia* (F) [84]				
*Aspergillus* (F) [84]	*Aspergillus* (F) [84]				
*Candida Albicans* (F) [82,83]	*Candida Albicans* (F) [82,83]				
*Malassezia Restricta* (T)(A) [81]	*Malassezia Restricta* (T)(A) [81]				
**Decrease**					
*Firmicutes* (T)(F) [57,58,63]	*Firmicutes* (T)(F) [57,63]				*Firmicutes* (F) [85]
*Erysipelotrichales* (T) [66] *					
*Bacteroidales* (T) [66] *					
*Clostridiales* (T) [66,67] *					
***Ruminococcaceae*** **(T)(F)** [63]	***Ruminococcaceae*** **(T)(F)** [63]	***Ruminococcaceae*** **(F****)** [18]	***Ruminococcaceae*** **(F****)** [18]		*Ruminococcaceae* (F) [85]
*Lachnospiraceae* (T) [63,65]	*Lachnospiraceae* (T) [63,65]	*Veillonellaceae* (T) [18]	*Veillonellaceae* (T) [18]		
*Fusobacteriacea* (F) [66] *		*Prevotellaceae* (T) [18]	*Prevotellaceae* (T) [18]		
*Neisseriaceae* (F) [66] *					
	***Clostridium*** **(F)** [19]	***Clostridium*** **(F)** [19]	***Clostridium*** **(F)** [19]		
***Ruminococcus*** **(T)** [63]	***Ruminococcus*** **(T)** [63]	***Ruminococcus*** **(F)** [22,89]	***Ruminococcus*** **(F)** [22]	***Ruminococcus*** **(F)** [89]	
***Akkermansia*** **(T)** [89]	***Akkermansia*** **(T)** [89]	***Akkermansia*** **(F)** [89]		***Akkermansia*** **(F)** [89]	
***Faecalibacterium*** **(T)(F)** [63,65,68,69]	***Faecalibacterium*** **(T)(F)** [63,65,68]	***Faecalibacterium*** **(F)** [22]	***Faecalibacterium*** **(F)** [22]		*Faecalibacterium* (F) [85]
*Roseburia* (T) [63,64,65]	*Roseburia* (T) [63,64,65]	*Lachnospira* (F) [22]	*Lachnospira* (F) [22]		
*Eubacterium* (T) [66] *					
*Dorea* (T)(F) [66] *					
*Blautia* (T) [66] *					
*Collinsella* (T) [63,65]	*Collinsella* (T) [63,65]				
*Coprococcus* (T) [66] *					
*Phascolarctobacterium* (T) [63]	*Phascolarctobacterium* (T) [63]				
***Faecalibacterium Prausnitzii*** **(T)** [63,66,69] *	***Faecalibacterium Prausnitzii*** **(T)** [19,63,64]	***Faecalibacterium Prausnitzii*** **(F)** [19,87]	***Faecalibacterium Prausnitzii*** **(F)** [19,87]		
	***Bifidobacterium Adolescentis*** **(F)** [64]	***Bifidobacterium Adolescentis*** **(F)** [20] ^#^	***Bifidobacterium Adolescentis*** **(F)** [20] ^#^		
*Coprococcus Comes* (F) [66] *		*Ruminococcus Obeum* (F) [87]	*Ruminococcus Obeum* (F) [87]		
*Bifidobacterium Longum* (F) [72]	*Bifidobacterium Longum* (F) [72]	*Eubacterium Hallii* (F) [87]	*Eubacterium Hallii* (F) [87]		
		*Eubacterium biforme* (F)(CAL) [86] ^#^			
		*Coprococcus Catus* (F)(CAL) [20,86,87] ^#^	*Coprococcus Catus* (F)(CAL) [20,87]		
		*Coprococcus Eutactus* (F) [87]	*Coprococcus Eutactus* (F) [87]		
		*Lachnospiraceae b. 5 1 63FAA* (F) [20] ^#^			
		*Roseburia Inulinivorans* (F) [20] ^#^	*Roseburia Inulinivorans* (F) [20] ^#^		
		*Prevotella copri* (F) [20] ^#^	*Prevotella copri* (F) [20] ^#^		
		*Dialister Invisus* (F) [20] ^#^	*Dialister Invisus* (F) [20] ^#^		
		*Clostridium IV* (F) [22]	*Clostridium IV* (F) [22]		
		*Clostridium XlVb* (F) [22]	*Clostridium XlVb* (F) [22]		
*Saccharomyces cerevisae* (F) [82,83]	*Saccharomyces cerevisae* (F) [82,83]				
*Candida tropicalis* (F) [84]	*Candida tropicalis* (F) [84]				
*Anelloviridae* (T) [79]	*Anelloviridae* (T) [79]				

Taxonomy of gut microbiota in IBD and SpA compared. Legend: 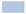 phylum, 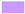 order, 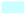 family, 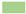 genus, 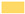 species, 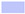 fungi, 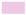 virus, (F) human fecal samples, (T) human tissue samples, (CAL) colonoscopy aspiration lavages, (A) animal models, * children population, ^#^ biological treatment. The highlighted bacteria are those whose intestinal concentration is altered in both diseases.

**Table 2:** Microbiota and TNFi therapy.

Work	Objective	Population	Result
Yin et al. [20]	Impact of TNFi on gut microbiota in AS.	67 AS treated with TNFi. 60 AS not treated with TNFi. 123 healthy controls.	Change in the microbiota after TNFi therapy Increase: *Bilophila, P. Copri, F. Prausnitzii, K. Pneumoniae, R. Bromii, Eubacterium Biforme*.
Dai et al. [93]	Impact of TNFi on gut microbiota in AS.	11 healthy controls. 24 AS before and after treatment with TNFi.	Change in the microbiota after TNFi therapy Increase: *Megamonas and Lachnoclostridium* (SCFA-producing bacteria)
Ditto et al. [94]	Impact of TNFi on gut microbiota in IBD-related SpA.	20 IBD-related SpA treated with TNFi.	Change in the microbiota after TNFi therapy Increase: *Lachnospiraceae, Clostridia, Lactobacillus, Coprococcus, R. Gnavus* Decrease: *Proteobacteria, Gammaproteobacteria*
Bazin et al. [95]	1. Impact of TNFi on gut microbiota in IBD-related SpA. 2. Research for bacteria predictive of the treatment with TNFi outcome in IBD-related SpA.	19 SpA treated with TNFi.	Diversity restored, but no significant differences in the concentration of any bacteria; *Burkholderiales* as a predictor of responder to TNFi.
Vallier et al. [96]	Research for bacteria predictive of the treatment outcome in AxSpA.	61 AxSpA treated with TNFi.	*Sutterella* and *Clostridia* as predictor of non-responder to TNFi.
